# A Descriptive Review of the Prevalence and Usage of Traditional and Complementary Medicine among Saudi Diabetic Patients

**DOI:** 10.1155/2018/6303190

**Published:** 2018-08-29

**Authors:** Saud Alsanad, Tamer Aboushanab, Mohammed Khalil, Osama A. Alkhamees

**Affiliations:** ^1^National Center for Complementary and Alternative Medicine, Ministry of Health, Riyadh, Saudi Arabia; ^2^College of Medicine, Al Imam Mohammad Ibn Saud Islamic University (IMSIU), Al-Nada, Riyadh 13317-4233, Saudi Arabia

## Abstract

Diabetes mellitus represents a major burden in Saudi Arabia where seven million (20% of population) are living with diabetes. This article reviews the literature on usage of traditional and complementary medicine (T&CM) therapies among Saudi diabetic patients, focusing in particular on identifying the prevalence as well as discussing their safety and efficacy. Three databases (PubMed, Cochrane, and ScienceDirect) were searched prior to December 10, 2017, for articles published in peer-reviewed journals that reported primary data on the use of traditional and complementary medicine therapies among diabetic patients in Saudi Arabia. Six studies were selected according to the inclusion/exclusion criteria. In conclusion, the prevalence of use of T&CM therapies among diabetics in Saudi Arabia was 32.18%. This review identified that the most used T&CM therapy among diabetics was herbal treatment. The most used herbs were fenugreek, black seeds, neem, myrrh, helteet, harmel, and aloes. There is insufficient or little evidence to support the efficacy of the most identified herbs and therapies. This review is raising the safety concerns about the used herbs and complementary therapies which were commonly used without any medical consultation.

## 1. Introduction

Diabetes mellitus (DM) is one of the most prevalent chronic diseases worldwide. The world prevalence of DM was estimated to be 285 million in 2010 which is predicted to increase to 485 million representing 7.7% of world adult population between 20–79 years by 2030 [1]. In 2012, 1.5 million deaths were due to DM [2].

In Saudi Arabia, the prevalence of diabetes is at an alarming level. Seven million (about 20% of the population) in Saudi Arabia are living with diabetes as estimated and reported by the World Health Organization (WHO) [3]. Saudi Arabia was the seventh in the world for the alarming rate of diabetes [4].

Complementary medicine practices have many definitions. The World Health Organization (WHO) published two strategies for traditional medicine. The WHO defined the term “traditional and complementary medicine.” Traditional medicine is the total knowledge of health-related practices and skills based on indigenous beliefs and experiences, while complementary medicine is the various health-related practices that are not part of that country's own tradition or conventional medicine [5]. The National Center for Complementary and Integrative Health (NCCIH) defined complementary therapies as health approaches outside of the mainstream western medicine. Recently, the NCCIH categorized complementary medicine into three categories: natural products, mind and body practices, and other complementary health approaches [6].

T&CM is widely used worldwide including Saudi Arabia. It was used mostly by people with chronic illnesses such as DM [5]. The prevalence of use of T&CM among diabetic patients globally was between 18% and 72.8% as reported by eighteen studies from nine countries. The most used complementary therapies were herbs, nutritional supplements, nutritional counseling, spiritual healing, and relaxation techniques [7]. In the USA, the prevalence was 57%, and the common complementary therapies used were religious and spiritual healing, herbs, relaxation and meditation techniques, chiropractic, and massage [8]. In India, the percentage of use was 67.8%, and the commonly used complementary therapies among diabetic patients were acupressure and naturopathy [9]. Evidently, the prevalence in Nigeria was 46%, and the herbal treatment was the most common therapy. The most used herbs were garlic (*Allium sativum*), ginger (*Zingiber officinale*), aloes (*Aloe vera*), and bitter leaf (*Vernonia amygdalina*) [10]. Furthermore, the prevalence of use in Malaysia was 62.5%. The most used therapies were biological therapy followed by manipulative-body-based systems. The most used herbs were bitter gourd (*Momordica charantia*), misai kucing (*Orthosiphon stamineus* Benth), garlic (*Allium sativum*), and sabah snake grass (*Clinacanthus nutans* Lindau) [11].

In the Gulf states, the prevalence of T&CM use is high. In Bahrain, 64% of diabetics reported using T&CM. The most used T&CM practices were natural medicines such as herbs, alternative medicine practices, and manipulative therapies. Garlic (*Allium sativum*), bitter melon (*Momordica charantia*), cinnamon (*Cinnamomum cassia*), and fenugreek (*Trigonella foenum-graecum*) were the most used herbs [12]. In Oman, the prevalence of use of T&CM among diabetics was 42%. The most used T&CM therapies were herbs and/or nutritional supplements. The most used herbs were harmel (*Rhazya stricta*), fenugreek (*Trigonella foenum-graecum*), and black seeds (*Nigella sativa*) [13]. In Iraq, the prevalence of use of herbs among diabetics was estimated to be 17.3%. Cinnamon, black seeds (*Nigella sativa*), and garlic (*Allium sativum*) were the most used herbs [14]. In Jordan, the prevalence of use of herbs was 31%. The most used herbs were fenugreek (*Trigonella foenum-graecum*), white lupine (*Lupinus albus*), garlic (*Allium sativum*), onion (*Allium cepa*), and black seeds (*Nigella sativa*) [15]. Another study showed that the prevalence of use was 16.6% and the most used herb was green tea [16]. In Lebanon, 38% was the prevalence of use of T&CM among diabetics. The most used T&CM practices were herbs and natural health products [17]. In summary, regional use of T&CM therapies among diabetic patients was between 17.3% and 64%. Herbal treatment was the most used T&CM practice among diabetics in Arabic and Gulf countries. The most used herbs were fenugreek (*Trigonella foenum-graecum*), garlic (*Allium sativum*), black seeds (*Nigella sativa*), cinnamon (*Cinnamomum cassia*), and bitter melon (*Momordica charantia*).

The current situation of the use of T&CM therapies among diabetic patients in Saudi Arabia was not evaluated extensively. This review is a step for prioritizing further research and programs for diabetic patients in Saudi Arabia. To the best of our knowledge, this is the first review to analyze the prevalence of use of T&CM therapies among DM patients in Saudi Arabia and discuss them against the available background information, safety, and effectiveness rating.

## 2. Methods

### 2.1. Literature Search Strategy

We searched PubMed, Cochrane, and ScienceDirect since inception until December 10, 2017, for articles published in peer-reviewed journals that report primary data on the use of T&CM among diabetic patients in Saudi Arabia.

We used a broad search strategy as we were expecting few studies to be available for the search question. The search terms used were “Saudi Arabia,” “complementary medicine,” “diabetes,” and “traditional medicine.” The search strategy was modified according to the database searched. Also, references included in full-text articles were searched manually.

### 2.2. Inclusion Criteria

Studies were included if they reported the prevalence and types of T&CM use among diabetic patients in Saudi Arabia, in English language, and full text was available.

### 2.3. Exclusion Criteria

Exclusion criteria were the following: non-English studies, data for diabetes could not be separated from other illnesses, and full-text articles cannot be retrieved.

### 2.4. Outcome

The main outcome of this review was to identify the prevalence and the most used therapies of T&CM among diabetics in Saudi Arabia. The prevalent therapies were discussed against the available background information, safety, and effectiveness rating.

### 2.5. Study Selection, Quality Assessment, and Data Extraction

Two reviewers Tamer Aboushanab and Mohammed Khalil (TA and MK) applied the criteria independently to the results of the searches. To assess the quality of the included studies, we used a quality assessment tool (QAT) developed by Bishop et al. which is based on the STROBE statement. The quality assessments included four domains: study design, sampling, participants' characteristics, and T&CM definition and use [18]. Data were extracted independently.

### 2.6. Statistical Analysis

To summarize the prevalence in different studies, we used a quantile around the measure of central tendency. However, a pooled estimate of prevalence was not used as it will ignore the variability in the study definition and design. Also, meta-analysis was not attempted due to the variation in definitions used, inaccessible raw data, and the heterogeneous methods of the included studies [19].

## 3. Results and Discussion

**Figure 1:**
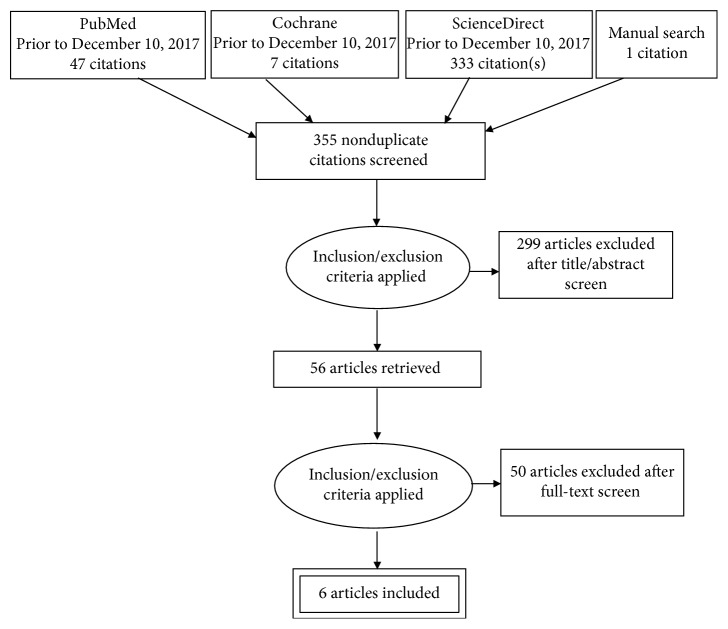
Search results (PRISMA).

Three hundred eighty-eight articles were retrieved through the database search (387) and manual search (1). The retrieved articles were reviewed by two independent researchers (TA and MK) according to the inclusion/exclusion criteria. 33 studies were excluded for duplication. 299 studies were excluded after title/abstract screening. 56 full-text articles were retrieved for further evaluation. 50 articles were excluded due to irrelevant information, to end with six studies. Total included studies were six studies (Figure 1). A summary of selected articles is shown in Table 1.

Al-Eidi et al. used the broad definition of T&CM and reported a prevalence of 92/302 (30.5%) (CI 25.42; 36) among type two diabetes mellitus patients. The most used T&CM therapies were herbs (30.4%), wet cupping (20.9%), nutritional supplements (17.6%), cautery (16.7%), spiritual healing (ruqia) (10.8%), apitherapy (2%), and massage (1.5%). [20] More than 78% of the T&CM users did not tell their physicians about the use of T&CM [20].

Al-Rowais studied specifically herbal use among diabetics, and she reported a prevalence of 51/296 (17.4%). The most used herbs were myrrh (*Commiphora molmol*), black seeds (*Nigella sativa*), fenugreek (*Trigonella foenum-graecum*), helteet (*Ferula assa-foetida*), and aloes (*Aloe vera*) [21]. About 73% of the T&CM users did not tell their physicians regarding the use of T&CM because physicians did not ask them about their use of T&CM modalities [21].

Al Saeedi also reported that the prevalence of use of traditional medicine remedies in Mecca, Saudi Arabia, among diabetics was 313/1039 (30.1%). The most used herbs were fenugreek (*Trigonella foenum-graecum*), chinaberry leaves (neem) (*Melia azedarach*), and harmel (*Rhazya stricta*) [22]. More than 70% of the T&CM users did not tell their physicians about the use of T&CM modalities, indicating an inadequate doctor-patient relationship [22].

Al-Garni et al. reported the prevalence of use of T&CM therapies among diabetics in Jeddah, Saudi Arabia, was 80/310 (25.8%). The most used herbs were ginger (*Zingiber officinale*), black seeds (*Nigella sativa*), and cinnamon (*Cinnamomum verum*) [23].

Kamel et al. reported a prevalence of 64% (137/214) among Saudi diabetics in the Jeddah city. This study included the smallest sample size among the six included studies (*n* = 214) [24]. The majority of the T&CM users in this study reported that they have not told their physicians about the use of T&CM and just a small number of physicians who asked them about their use [24].

Bakhotmah and Alzahrani included only patients who are suffering from foot disorders. The prevalence of T&CM use among these diabetic patients was 34.4% (142/1006), who used CM alone, and 204/1006, who used CM and conventional treatment. Honey, myrrh (*Commiphora molmol*), black seeds (*Nigella sativa*), fenugreek (*Trigonella foenum-graecum*), and henna (*Lawsonia inermis*) were the most used natural treatments. Honey with black seeds (*Nigella sativa*) followed by honey with myrrh (*Commiphora molmol*) combinations were the most used topical combinations [25]. About 75% of the T&CM users did not consult their doctors before the use of T&CM therapies [25].

A total of 3167 diabetic patients were surveyed in all selected studies. The prevalence of use of T&CM therapies among diabetic patients in all studies was 32.18% (1019/3167) (CI 30.56; 33.84). The most used CM therapies were herbs and honey. The most used herbs were fenugreek, black seeds, neem, myrrh, helteet, harmel, and aloes. This is our first outcome measure of this review. However, different definitions and groups of T&CM therapies and different questionnaires were used. This may explain the wide range of the prevalence of use from 17.4% to 64%.

Out of the six included studies, 4 were conducted in the western region of Saudi Arabia, 3 in the Jeddah city [23–25] and one in the Mecca city [22]. The remaining two were in the Riyadh city [20, 21]. Five studies were conducted in the outpatient clinics [20–24], and the remaining one used a household design [25]. Only one study used the broad definition of T&CM [20], while the remaining concentrated mainly on herbs. The majority of T&CM users did not tell their physicians about the use of T&CM therapies [20–22, 24, 25]. And the majority of physicians did not ask their patients about their use of T&CM therapies [21, 22, 24]. The quality assessment of the included studies ranged from low to medium quality (50% to 72.2%) with the overall assessment of 65.3%.

In summary, the prevalence of T&CM use in Saudi Arabia was between 17.4% and 64%. The prevalence of use of T&CM therapies among diabetic patients in all studies in Saudi Arabia was 32.18% as estimated by this review. The most used T&CM therapies were herbs and honey.

As a comparison between the prevalence of use of T&CM among diabetics in Saudi Arabia and other countries, the prevalence in Saudi Arabia is about half the prevalence in countries such as India (67.8%) [9] and Malaysia (62.5%) [11]. The prevalence of use of T&CM among diabetics in Saudi Arabia comes fourth after Bahrain (64%) [12], Oman (42%) [13], and Lebanon (38%) [17] among Arabic countries. There is a lack of information about the prevalence of use of T&CM among diabetics in most of the Arabic countries.

## 4. The Most Used T&CM Therapies in Saudi Arabia

The Natural Medicines Comprehensive Database is one of the most reliable and completed herbal and nonherbal resources available for consumers and healthcare professionals [26]. The database has an evidence-based effectiveness rating, which was categorized into the following seven categories: “effective, likely effective, possibly effective, possibly ineffective, likely ineffective, ineffective, and insufficient evidence.” Safety rating was categorized into the following six categories: “safe, likely safe, possibly safe, possibly unsafe, likely unsafe, and unsafe” [27]. The Natural Medicines Comprehensive Database was the main base of assessment. The most used herbs by diabetics in Saudi Arabia and their effectiveness and safety rating according to the Natural Medicines Comprehensive Database are mentioned in Table 2. The most used complementary medicine practices other than herbs are mentioned in Table 3.

### 4.1. Herbs

#### 4.1.1. Black Seeds

Ancient Egyptians and Greeks prescribed black seeds or black cumin (*Nigella sativa*) for some ailments such as headache and to increase milk production. It was used traditionally in the Middle East for a variety of diseases such as asthma and hypertension [28]. In Arabian Gulf countries, black seeds were prescribed for a variety of ailments including diabetes [29]. One study suggested that 2 grams of black seeds per day besides antidiabetic medications could significantly improve the results of the fasting blood glucose test, two-hour postprandial glucose test, and glycosylated hemoglobin test in patients with type 2 diabetes [30]. In animal studies, significant benefits of black seeds for diabetic animals were reported [31]. Further large-scale, randomized clinical trials are recommended to confirm the results. Black seeds in therapeutic doses had a wide safety margin [32].

#### 4.1.2. Fenugreek

Fenugreek (*Trigonella foenum-graecum*) is a traditional herb used for thousands of years by various traditional medical systems such as Ayurveda to treat diabetes mellitus [33]. Fenugreek may have positive effects by promotion of insulin secretion effects and enhancement of peripheral utilization of glucose [34]. Various studies reported the benefits and the wide safety margin of fenugreek [32].

#### 4.1.3. Myrrh

Myrrh (*Commiphora myrrha*) is an old traditional medicinal herb. Myrrh was used by ancient Egyptians. The word “myrrh” was extracted from the Arabic word “mur” which means “bitter” [34]. Myrrh may have an antiglycemic and antioxidant effect on animals [35]. It may also be used locally with or without honey for the treatment of wounds even in diabetic patients but for short periods (less than two weeks) and low concentration as it seemed to have harmful adverse events if used in high concentrations or long periods [36, 37].

#### 4.1.4. Helteet

Helteet (*Ferula assa-foetida*) is a traditional spice and a medicinal herb. Helteet is used traditionally as an anthelmintic, antispasmodic, and antidiabetic herb [38]. In animal studies, there is preliminary evidence suggesting the potential antihyperglycemic effect of helteet [39]. It had a good toxicity profile for a dose of 250 mg/kg for a short period on animals [40].

#### 4.1.5. Neem

Neem (*Melia azedarach*) may have hypoglycemic properties. Leaves, bark, stem, and seed oil are the used parts of neem [41]. In animal studies, neem (*Melia azedarach*) has potential benefits for the treatment of diabetes with or without other herbs such as African bitter leaf [41, 42]. Interestingly, tulsi (holy basil) (*Ocimum sanctum*) and neem (*Azadirachta indica*) had a significant improvement in diabetic symptoms in male diabetic patients [43]. Neem (*Melia azedarach*) poisoning was reported. High doses of neem may lead to serious adverse events or death [44].

#### 4.1.6. Harmel

Harmel (*Rhazya stricta*) is cultivated in the Arabic region and used as a folk medicine. Harmel is used traditionally in the treatment of diabetes mellitus, sore throat, and inflammatory conditions [45]. Animal studies showed controversial results. Wasfi et al. reported no significant results [46]. However, Ali reported a significant decrease in the blood glucose level and an increase of insulin when using harmel and glibenclamide [47]. Adverse events of harmel were reported in animals which included the death of a sheep. Harmel may interact with antidiabetic drugs [48].

#### 4.1.7. Aloes

Aloe (*Aloe vera*) is cultivated in North Africa and Turkey. It is used traditionally as a laxative, anti-inflammatory agent and in the treatment of wounds and burns. However, controversial results of antidiabetic effects of aloe were reported which may be due to the use of different parts of the plant [49]. Aloe showed antioxidant and antihyperlipidemia effects in animal studies [50, 51]. Oral administration of aloe may cause numerous adverse events such as diarrhea, drug interaction, and kidney dysfunction, while adverse events of local application of aloe may include dermatitis, photoirritation, and erythema [52].

#### 4.1.8. Ginger

Ginger (*Zingiber officinale*) is a traditional medicinal herb and a popular spice. Ginger is used for more than 2500 years [53]. Mozaffari-Khosravi et al. suggested that the use of 3 grams of ginger for eight weeks may be beneficial for diabetic patients and may lower both fasting blood sugar and glycosylated hemoglobin [53]. Ginger was also considered relatively safe [54].

#### 4.1.9. Cinnamon

Common cinnamon (*Cinnamomum verum* and *Cinnamomum zeylanicum*) and cassia cinnamon (*Cinnamomum aromaticum*) were popular worldwide flavoring herbs. Cinnamon was used for more than 4000 years [55]. Khan et al. suggested that the use of 1 to 6 grams/day of cinnamon could significantly reduce blood glucose, triglycerides, LDL and total cholesterol [56]. Cinnamon (common and cassia) seemed to be relative safe and a well-tolerated herb. Allergy and contact reactions were the most reported adverse events [55].

### 4.2. Other Complementary Therapies

#### 4.2.1. Honey

Honey was used locally and orally for the treatment of diseases. Studies showed the antioxidant and dose-dependent hypoglycemic effects of honey [57]. Bahrami et al. reported that the consumption of honey for 8 weeks may increase slightly the hemoglobin A1c, which indicated an increase in blood glucose level and decrease in the body weight and blood lipids [58]. Honey had a wide safety margin except for infants less than one year because of reported cases of botulism [59].

#### 4.2.2. Wet Cupping

Cupping therapy is a physical therapy utilized by the complementary medicine practitioners. Wet cupping is a type of cupping therapy which is performed by making superficial skin incisions that lead to drawing blood into the applied cups [60]. Alshowafi reported the reduction of fasting blood glucose and triglycerides after wet cupping therapy [61]. Cupping therapy was relatively safe and should be practiced by qualified licensed therapists [62]. Currently, cupping therapy (Hijama in Arabic) is regulated by the National Center for Complementary and Alternative Medicine (NCCAM), Ministry of Health, Saudi Arabia.

#### 4.2.3. Nutritional Supplements (Vitamins/Minerals)

According to the American Diabetic Association, there is no sufficient or clear evidence to support the effectiveness of using vitamins and minerals to improve the condition of diabetic patients in the absence of vitamins or minerals deficiencies. Multiple safety concerns about the long-term use of antioxidants exist [63]. Suksomboon et al. concluded that the chromium had favorable effects for diabetics without an increase in adverse events if taken at usual doses [64]. Barbagallo and Dominguez reported the preliminary benefits of oral magnesium supplementation for diabetics and recommended conducting large-scale trials to confirm the results [65]. Chromium, magnesium, zinc, calcium. folic acid, vitamin B6, vitamin C, vitamin E, vitamin D, and vitamin K are examples of the used vitamins and minerals (Table 3).

#### 4.2.4. Cautery

Cautery (kaiy in Arabic) is one of the oldest ancient healing techniques without any scientific evidence. Traditional healers performed cautery by burning specific skin points according to the treated disease [66]. Severe complications were reported after cauterization. Wound infection, delayed healing, abscess formation, septic shock, and deep skin burn were examples [67].

#### 4.2.5. Spiritual Healing (Ruqia)

Spiritual healing is a systemic intervention or interaction between the healer and the patient with the aim to improve or cure the condition. 13000 spiritual healing practitioners have registered in the UK alone [68]. Ruqia is the recitation of special prayers. Al-Dalee and Aljubran reported that 92% of cancer patients in Saudi Arabia used Quran or ruqia as a complementary treatment [69].

#### 4.2.6. Apitherapy (Honeybee Products)

Apitherapy is the utilization of honey, propolis, royal jelly, bee venom, wax, and pollen for the treatment of diseases. The history of apitherapy was dated back to more than 6000 years [70]. Propolis was used to treat diabetes, and some animal studies reported its beneficial effects. Long-term treatment with propolis was beneficial in reducing complications in type 1 diabetes patients and decreasing fasting blood sugar in type 2 diabetes patients [71]. Allergic reactions were the most reported adverse events of propolis. In Italy, eighteen adverse events cases were recorded in 5 years by the Italian pharmacovigilance system: sixteen of them were allergic reactions and two cases were digestive ailments [72].

#### 4.2.7. Massage

Massage is a complementary body-based therapy. Massage is based on performing certain manipulations and movements such as friction, kneading, tapping, and cupping movements [73]. Ezzo et al. reported the increase of insulin absorption when using massage at the injection site for type 1 diabetic patients and the benefits of massage for diabetic neuropathy [73]. Massage is a relatively safe therapy due to the minor and low number of reported adverse events. Spinal manipulation as a type of massage was associated with the most serious adverse events such as disc herniation and spinal cord injury [74].

### 4.3. Study Limitations

The little number of studies and surveys conducted in Saudi Arabia to identify the prevalence and the pattern of use of complementary medicine among diabetic patients was one of the most important limitations. The small number of surveyed patients in retrieved studies was another limitation. The conducted studies did not represent the whole population as they were conducted in just three cities in Saudi Arabia (Riyadh, Jeddah, and Mecca). The lack of well-designed large-scale trials and insufficient evidence for most of the identified herbs was also an important limitation.

## 5. Conclusion

The prevalence of use of T&CM therapies and products is moderate to high among diabetics in Saudi Arabia. The estimated prevalence of use of T&CM therapies among diabetic patients in all selected studies was 32.18% (1019/3167). The most used CM therapies were herbs and honey. The most used herbs were fenugreek, black seeds, neem, myrrh, helteet, harmel, and aloes. Herbal medicine was the most used T&CM among diabetics locally, regionally, and worldwide. There is insufficient or little evidence to support the efficacy of the most identified herbs and therapies. This review is raising the safety concerns about the used herbs and complementary therapies which were commonly used without any medical consultation. The majority of T&CM users did not tell their physicians about the use of T&CM therapies. On the contrary, the majority of physicians did not ask their patients about their use of T&CM therapies. We recommend the following: (1) physicians should ask their diabetic patients about the use of T&CM therapies in every consultation, and this should be an essential part of any clinical follow-up. (2) Physicians should increase their knowledge regarding the use of T&CM therapies and products. (3) The orientation of T&CM therapies should be included in health promotion and education programs. The current review encourages further research on the potential interaction between the T&CM used in Saudi Arabia and modern medicine medications of DM treatment.

## Figures and Tables

**Table 1:** A summary of retrieved studies conducted in Saudi Arabia.

Author (year)	Mode of data collection	Setting	Sample size	Response rate	Males	Mean age	T&CM definition	Prevalence and CI	Condition	Types
Al-Garni et al. [23]	Outpatient cross-sectional study	Jeddah Diabetic Center (JDC), Jeddah	310	Not reported	75.2%	57.58 ± 8.50	Herbal and food supplements	25.8% (21.10; 31.12)	Type 2 diabetes	Herbal medicine (25.8%): ginger (*Zingiber officinale*) (11.6%), black seeds (*Nigella sativa*) (10%), cinnamon (*Cinnamomum verum*) (5.5%), fenugreek (*Trigonella foenum-graecum*) (2.9%), and garlic (*Allium sativum*) (2.9%), Food supplement (30.6%): B complex (15.2%), vitamin D (14.5%), and calcium (0.6%)

Al-Rowais [21]	Outpatient cross-sectional study	Riyadh city: outpatient clinics in King Khalid University, King Abdulaziz University Hospital, Prince Salman Hospital, and Riyadh Medical Complex	296	98.6%	56.7%	51.99 ± 15.6	Herbal	17.4% (13.36; 22.31)	Diabetes	Herbal medicine (17.4%): myrrh (*Commiphora molmol*) (45%), black seeds (*Nigella sativa*) (19.6%), helteet (*Ferula assa-foetida*) (13.7%), fenugreek (*Trigonella foenum-graecum*) (13.7%), and aloes (*Aloe vera*) (11.8%)

Al Saeedi et al. [22]	Outpatient cross-sectional study	Mecca city: 7 government hospitals plus private hospitals	1039	89.4%	66.7%	Not reported	Traditional remedies (herbal)	30.1% (27.34; 33.01)	Diabetes	Herbal medicine (30.1%): fenugreek (*Trigonella foenum-graecum*) (6.1%) and neem (*Melia azedarach*) (5.1%)

Bakhotmah and Alzahrani [25]	Household cross-sectional study	Jeddah city	1006	Not reported	53.1%	49 ± 17	Topical natural preparation	34.4% (31.48; 37.44)	Diabetic foot	Honey (56.6%), myrrh (*Commiphora molmol*) (37.4%), black seeds (*Nigella sativa*) (35.1%), fenugreek (*Trigonella foenum-graecum*) (12.5%), honey and black seeds combination (19.1%), and honey and myrrh combination (12.1%)

Al-Eidi et al. [20]	Outpatient cross-sectional study	Riyadh city: outpatient clinics in Diabetic Center of King Salman bin Abdulaziz Hospital	302	Not reported	43.4%	51.6 ± 10.6	All types	30.50% (25.42; 36.08)	Type 2 diabetes	Herbs (30.4%), wet cupping (20.9%), vitamins and minerals (17.6%), cautery (16.7%), ruqia (spiritual healing) (10.8%), honeybee products (2.0%), and medical massage (1.5%)

Kamel et al. [24]	Outpatient cross-sectional study	Jeddah city: King Abdulaziz University and King Fahad Hospital	214	71.3%	40%	Not reported	Herbal	64% (57.14; 70.35)	Diabetes	Herbs (64%)

**Table 2:** The most used herbs among diabetics in Saudi Arabia, with their effectiveness and safety rating being extracted from the “Natural Medicines Comprehensive Database.”

Number	Common name	Scientific name	Effectiveness rating	Safety rating for adults	Safety rating during pregnancy
1	Black seeds	*Nigella sativa*	Insufficient evidence	Likely safe	Likely unsafe for high doses
2	Fenugreek	*Trigonella foenum-graecum*	Possibly effective	Likely safe	Likely safe
3	Myrrh	*Commiphora myrrha* (*Commiphora molmol*)	Insufficient evidence	Unsafe for high doses	Unsafe
4	Helteet	*Ferula assa-foetida*	Insufficient evidence	Possibly safe	Unsafe
5	Chinaberry leaves (neem)	*Melia azedarach*	Insufficient evidence	Possibly safe (low doses for short periods) and possibly unsafe (large doses or for long periods)	Likely unsafe
6	Harmel	*Rhazya stricta*	Insufficient evidence	Likely unsafe	Likely unsafe
7	Aloes	*Aloe vera*	Insufficient evidence	By mouth: possibly unsafe	Possibly unsafe
8	Ginger	*Zingiber officinale*	Insufficient evidence	Likely safe	Possibly safe
9	Cinnamon	*Cinnamomum verum*, *C. zeylanicum*	Possibly effective	Therapeutic doses: likely safe; high doses or for long periods: possibly unsafe	Insufficient information, so avoid

**Table 3:** The most used CAM among diabetics in Saudi Arabia, with effectiveness and safety rating being extracted from the “Natural Medicines Comprehensive Database.”

Number	Name	Effectiveness	Safety rating for adults	Safety rating during pregnancy
1	Honey	Insufficient evidence	Likely safe. Honey (from the nectar of *Rhododendron*) is likely unsafe	Likely safe in food amounts
2	Wet cupping	No information	No information	No information, so avoid
3	Nutritional supplements (vitamins/minerals)			
a	Chromium	Possibly effective	Likely safe	Likely safe. Must be prescribed by doctors
b	Magnesium	Possibly effective	Therapeutic doses: likely safe. High doses: possibly unsafe	Therapeutic doses: likely safe. High doses: possibly unsafe
c	Calcium	Insufficient evidence	Therapeutic doses: likely safe. High doses: possibly unsafe	Likely safe
d	Folic acid	Insufficient evidence	Therapeutic doses: likely safe. High doses: possibly unsafe	Likely safe
e	Zinc	Insufficient evidence	Less than 40 mg/day is likely safe. High doses: likely unsafe. Nasal route is possibly unsafe	Therapeutic doses: likely safe. High doses: likely unsafe
f	Vitamin B6	Insufficient evidence	Therapeutic doses: likely safe. Oral high doses: possibly unsafe	Therapeutic doses: likely safe. Oral high doses: unsafe
g	Vitamin C	Insufficient evidence	Therapeutic doses: likely safe. Doses more than 2000 mg/day are possibly unsafe	Therapeutic doses: likely safe. Doses more than 2000 mg/day are possibly unsafe
h	Vitamin D	Insufficient evidence	Therapeutic doses: likely safe. Doses more than 4000 units/day for long periods are possibly unsafe	Therapeutic doses less than 4000 units/day are likely safe. Higher doses are possibly unsafe
i	Vitamin E	Insufficient evidence	Recommended dose which is 22.4 IU/day: likely safe. High doses: possibly unsafe	Recommended dose which is 22.4 U/day is likely safe. Avoid in early pregnancy
j	Vitamin K	Insufficient evidence	Recommended daily dose: likely safe	Recommended daily dose: likely safe
4	Cautery	No information	No information	No information, so avoid
5	Spiritual healing (ruqia)	Insufficient evidence	Safe when used correctly, but it is not an alternative to usual medical care or other therapies	Safe when used correctly
6	Apitherapy (bee products)	Insufficient evidence	Safe if used appropriately. Bee venom must be practiced by licensed healthcare professionals	Unsafe in high doses
7	Massage	Insufficient evidence	Likely safe	Likely safe
